# Assessing Parental Preferences for Children's Oral Health in Rural Areas: A Study on Maintenance and Treatment Choices

**DOI:** 10.7759/cureus.59773

**Published:** 2024-05-06

**Authors:** Rahul Mishra, Gourav Jain, Atul K Singh, Bipin K Yadav, Rajesh Kumar Thakur, Ashutosh Dixit

**Affiliations:** 1 Pedodontics and Preventive Dentistry, Faculty of Dental Sciences, Uttar Pradesh University of Medical Sciences, Etawah, IND; 2 Oral and Maxillofacial Surgery, Faculty of Dental Sciences, Uttar Pradesh University of Medical Sciences, Etawah, IND; 3 Periodontology, Faculty of Dental Sciences, Uttar Pradesh University of Medical Sciences, Etawah, IND; 4 Dentistry, All India Institute of Medical Sciences, Rishikesh, Rishikesh, IND

**Keywords:** rural population, pediatric treatment modalities, parental education, parental acceptance, child oral health

## Abstract

Aim: This article investigates the oral health preferences of parents residing in rural areas for their children, including dental maintenance and treatment.

Materials and methods: Data were collected through a cross-sectional survey of 500 parents who sought dental consultation for their children between two and seven years old, excluding those with systemic or neurological disorders. Demographic characteristics, including child and parent age and gender, family structure, and parental education levels, were collected using self-administered questionnaires.

Results: The study on oral health preferences of rural parents reveals the following key trends: peak dental treatment interest at six years old (104 children), slight gender disparity favoring males (54.8%), prevalent joint family structures (58%), and families with two children (48%). Most mothers marry before age 21 (62.8%), with varied education levels. Toothpaste and toothbrushes are preferred by the majority (65.2%) for oral hygiene. Common treatments include oral prophylaxis (164), pulpectomy/pulpotomy (114), and extractions (86). However, significant gaps exist: 62.8% do not grasp the importance of primary teeth, and 72% lack knowledge about specific treatments and drug-induced tooth problems, urging targeted educational strategies.

Conclusion: The study's outcome focuses on parental preferences for oral hygiene maintenance and their choices for dental treatment in primary dentition. The results highlight the influence of various factors on parental preferences and underscore the importance of improving parental knowledge for better oral health outcomes in rural populations.

## Introduction

Oral health plays a pivotal role in children's overall well-being, impacting not only their physical health but also their social and psychological development. In rural populations, where access to dental care resources can be limited, the importance of parental preferences and attitudes toward oral hygiene and dental treatment becomes even more pronounced. This article aims to delve into the intricate dynamics of parental preferences regarding oral health maintenance and dental care for children residing in rural areas [1,2].

Rural populations often face unique challenges in accessing quality dental care services. Factors such as geographical remoteness, economic disparities, and a shortage of dental professionals contribute to this healthcare disparity [3]. Consequently, parents in rural settings may be compelled to make distinctive choices regarding their children's oral health. Understanding these choices, the factors influencing them, and their potential consequences is vital for designing effective oral health interventions tailored to rural communities.

The influence of parental preferences on oral hygiene practices and dental treatment-seeking behaviors in rural areas is multifaceted. It encompasses considerations related to cultural norms, socioeconomic status, knowledge, and available healthcare infrastructure. By comprehensively evaluating these preferences, this study aims to shed light on the underlying factors that shape oral health behaviors in rural families [1,3]. Moreover, it seeks to identify opportunities for targeted interventions that can improve oral hygiene practices and increase access to dental care services for children in these underserved regions [4,5].

This article explores the methodologies employed for assessing parental preferences regarding oral health in rural populations, detailing the data collection processes and subsequent analysis. It seeks to answer questions surrounding how parental preferences were evaluated and interpreted in the context of oral health interventions. Furthermore, the article explores the potential implications of these findings for promoting oral health and designing effective intervention strategies tailored to rural communities. Ultimately, the research endeavors to provide valuable insights that can inform the development of policies and programs aimed at enhancing oral health outcomes among children living in rural areas.

## Materials and methods

Ethical approval for this study was obtained from the Research Ethics Committee of the Faculty of Dental Sciences, Uttar Pradesh University of Medical Sciences, Saifai, Etawah, with institutional review board number UPUMS/2020/176. The study was conducted from June 2021 to May 2022. Informed consent was secured from all participating parents. During the informed consent process, the researchers were available to address any questions or concerns raised by the parents. This allowed for open communication and ensured that the parents were fully informed before deciding on participation. Once the parents indicated their willingness to participate by signing the informed consent form, they were included in the study. Throughout the data collection process, the researchers paid attention to the participants' needs and concerns, providing support and reassurance as needed. This approach helped establish trust and rapport with the parents, facilitating their engagement in the study and ensuring the ethical conduct of the research. The study involved a cross-sectional survey of 500 parents of children between two and seven years old who were seeking dental consultation in the department. Children with systemic diseases or neurological disorders were excluded from the study.

Inclusion criteria included parents of healthy children aged two to seven years who were seeking dental consultation in the department of the Faculty of Dental Sciences at Uttar Pradesh University of Medical Sciences, Saifai, Etawah. Those who were willing to sign the informed consent were included as participants in the study. By including parents of children within this age group, the study aimed to gather insights into parental preferences and practices regarding oral hygiene maintenance and dental treatment during early childhood. Exclusion criteria included parents who were unwilling to participate in this study.

Data were collected using the nonprobability sampling method and self-administered questionnaires completed by the parents who spent most of the time with the child. The questionnaire was developed based on a comprehensive review of existing literature and relevant theoretical frameworks related to parental attitudes and practices toward children's oral health. To ensure the robustness and credibility of our findings, extensive validity and reliability assessments were conducted on the questionnaire used in this study. Content validity was established through expert reviews from seasoned dental health professionals who evaluated the questionnaire items for their relevance and comprehensiveness in capturing the various aspects of parental preferences regarding children’s oral health. The face value obtained was 1. Additionally, test-retest reliability was assessed by administering the questionnaire to a small subset of the target population at two different time points. The consistency of responses obtained confirmed the reliability of the instrument, thereby ensuring that the data collected were stable over time and reflective of true parental attitudes and behaviors. The Cronbach's alpha value was 0.8.

The study was planned meticulously, which included specifying the purposive nonprobability sampling method used for participant selection, allowing us to focus on a specific demographic of parents of children aged two to seven years seeking dental consultation. We also provided a comprehensive breakdown of the demographic characteristics collected, which included not only the age and education level of the parents but also detailed information on family structure and socioeconomic status. Furthermore, the administration of the questionnaire was conducted under controlled settings to minimize external influences, and the data were anonymized and securely stored to uphold participant confidentiality and data integrity. These steps were taken to ensure that the study adhered to the highest standards of research ethics and methodology. The self-administered questionnaire used for the study is displayed in Table 1.

**Table 1:** Self-administered questionnaires for the study

Questionnaire for assessing parental preferences for children's oral health in rural areas based on the criteria provided
1. What is the age of your child?
a. 2 years
b. 3 years
c. 4 years
d. 5 years
e. 6 years
f. 7 years
2. What is the gender of your child?
a. Male
b. Female
3. What is the structure of your family?
a. Nuclear family
b. Joint family
4. How many children are there in your family?
5. What was the age of the mother at the time of marriage?
6. What is the father's education level?
a. Uneducated
b. Up to 5th class education
c. Class 6th-8th education
d. Class 9th-12th education
e. Graduate
f. Postgraduate
7. What is the mother's education level?
a. Uneducated
b. Up to 5th class education
c. Class 6th-8th education
d. Class 9th-12th education
e. Graduate
f. Postgraduate
8. i) How important do you consider oral hygiene maintenance for your child?
a. Not important
b. Somewhat important
c. Important
d. Very important
ii) Means for oral hygiene maintenance
a. Using a herbal stick like neem
b. Using toothpaste and toothbrush
c. Using other means of oral hygiene maintenance
9. What are your preferences for various dental treatments for your child?
a. Regular checkups
b. Oral prophylaxis
c. Dental sealants
d. Fillings
e. Pulpectomy/pulpotomy
f. Extractions
g. Stainless steel crown placement
h. Others (please specify)
10. How knowledgeable are you about the importance of primary teeth?
a. Not knowledgeable
b. Somewhat knowledgeable
c. Knowledgeable
11. How knowledgeable are you about specific treatments for dental problems?
a. Not knowledgeable
b. Knowledgeable
12. How knowledgeable are you about drug-induced tooth problems?
a. Not knowledgeable
b. Somewhat knowledgeable
c. Knowledgeable
d. Very knowledgeable

The questionnaires gathered information on demographic characteristics, including the age and gender of both the child and the parent, family structure (nuclear or joint), and the education level of both mother and father. The primary outcome of the study focused on parental preferences regarding oral hygiene maintenance and their choices for dental treatment in cases of primary dentition issues. The descriptive analysis was done to determine the statistical analysis.

## Results

The demographical data of the individuals are given in Table 2.

**Table 2:** Demographic data

Demographic variable	Number of participants
Child’s age	
2 years	70
3 years	79
4 years	84
5 years	96
6 years	104
7 years	67
Child’s gender	
Male	274
Female	226
Family structure	
Joint family	290
Nuclear family	210
Number of children in the family	
One	120
Two	240
More than two	140
Mother’s age at marriage	
Less than 21 years	314
More than 21 years	186
Father’s education	
Uneducated	50
Up to 5th class	140
6th-8th class	180
9th-12th class	80
Graduate	30
Postgraduate	20
Mother’s education	
Uneducated	140
Up to 5th class	160
6th-8th class	110
9th-12th class	60
Graduate	20
Postgraduate	10

The study revealed a correlation between a child's age and an increase in parental engagement in dental treatment and oral hygiene maintenance, with the highest demand observed among six-year-olds. Furthermore, a slight gender imbalance was noted, with more males seeking dental treatment than females. Family structure also played a role, with a significant proportion living in joint families, potentially influencing oral health decision-making. Additionally, families with two children constituted the largest group, indicating potential variations in oral health practices based on family size. Moreover, a substantial majority of mothers were married before the age of 21, suggesting potential implications for their awareness and practices related to children's oral health. These insights highlight the importance of considering demographic factors in designing oral health interventions tailored to rural communities. The descriptive analysis of the results is shown in Table 3.

**Table 3:** Descriptive analysis of the results

Variables	N (%)
According to the child’s age	
2 years	70 (14%)
3 years	79 (15.8%)
4 years	84 (16.8%)
5 years	96 (19.2%)
6 years	104 (20.8%)
7 years	67 (13.4%)
According to the child’s gender	
Male	274 (54.8%)
Female	226 (45.2%)
According to the family structure	
Joint family	290 (58%)
Nuclear family	210 (42%)
According to the number of children in the family	
One	120 (24%)
Two	240 (48%)
More than two	140 (28%)
According to the mother’s age at the time of marriage	
Less than 21 years	314 (62.8%)
More than 21 years	186 (37.2%)
According to the father’s education	
Uneducated	50 (10%)
Up to 5th class	140 (28%)
6th-8th class	180 (36%)
9th-12th class	80 (16%)
Graduate	30 (6%)
Postgraduate	20 (4%)
According to the mother’s education	
Uneducated	140 (36%)
Up to 5th class	160 (14%)
6th-8th class	110 (22%)
9th-12th class	60 (12%)
Graduate	20 (4%)
Postgraduate	10 (2%)
According to parental preference for oral hygiene maintenance	
Using toothpaste and toothbrush	326 (65.2%)
Using a herbal stick like neem	108 (21.6%)
Using other means of oral hygiene maintenance	44 (8.8%)
No means of oral hygiene maintenance	22 (4.4%)
According to parental preference for various dental treatment	
Oral prophylaxis	164 (32.8%)
Dental restoration	44 (8.8%)
Extraction	86 (17.2%)
Pulpectomy/pulpotomy	114 (22.8%)
Stainless steel crown placement	18 (3.6%)
No treatment	74 (14.8%)
According to parents' knowledge about the importance of primary teeth	
Yes	186 (37.2%)
No	314 (62.8%)
According to parents' knowledge about specific treatment for dental problem	
Yes	140 (28%)
No	360 (72%)
According to parents' knowledge about drug-induced tooth problems	
Yes	140 (28%)
No	360 (72%)

These insights suggest that there is a potential gap in oral health knowledge and practices among parents in rural areas, especially regarding the importance of primary teeth and specific dental treatments. Tailored educational programs and interventions could help improve oral health awareness and practices in these communities.

Inferential statistical analysis was employed to assess the relationships between various demographic and behavioral variables. A significant relationship was found between the child’s gender and oral hygiene practices, suggesting that gender may influence how oral hygiene is maintained. Additionally, the age of the mother at the time of marriage was significantly associated with the choice of dental treatments, indicating that earlier family responsibilities might influence health decisions. Parents' education levels showed a significant correlation with their knowledge about the importance of primary teeth, highlighting the role of education in health literacy. Finally, the family structure was significantly related to treatment preferences, suggesting that the type of family (nuclear vs. joint) influences dental care decisions. These findings suggest that targeted educational and intervention strategies should consider these demographic factors to enhance oral health outcomes (Table 4).

**Table 4:** Inferential statistics for the comparisons in the group of participants

Variable	Chi-square/t-test	p value	Interpretation
Child’s gender vs. oral hygiene	χ² = 8.76	p = 0.03	Statistically significant
Age of mother at marriage vs. dental treatments	t = 2.45	p = 0.014	Statistically significant
Parent’s education level vs. knowledge about primary teeth	χ² = 12.42	p = 0.002	Statistically significant
Family structure vs. treatment preferences	χ² = 5.67	p = 0.017	Statistically significant

## Discussion

Oral health is a fundamental component of overall well-being, particularly during childhood, when lifelong dental habits are established [2]. Parental preferences and choices regarding oral hygiene maintenance and dental treatment play a pivotal role in shaping a child's oral health outcomes. This study delves into the preferences of parents living in rural areas, shedding light on their attitudes toward oral health and pediatric dental care, which correlate with a study done by Singh et al. [1]. The findings provide valuable insights that can inform targeted interventions to improve oral health practices in rural populations [5]. The study indicates that parental preferences evolve as the child's age increases. As children grow older, they may encounter new dental issues or developmental milestones, prompting parents to adjust their preferences for oral hygiene maintenance and dental treatment accordingly. Moreover, as children become more independent, parents seek more proactive approaches to address oral health concerns and ensure optimal dental care for their growing child [4,6]. The prevalence of joint families in rural areas (290 respondents) may impact parental preferences. In such family structures, there may be a collective decision-making process regarding oral health practices. In contrast, nuclear families (210 respondents) may rely more on individual choices. Folayan et al. also stated that these dynamics can significantly influence the type of oral hygiene maintenance and dental treatments chosen [7].

Bennadi et al. [6] stated that mothers' increased education is directly related to a child’s good oral health status. The level of education among parents also played a significant role. The majority of parents had completed education up to the sixth to eighth grade, but there were variations in education levels. Higher education levels were often correlated with better awareness of oral health practices and a greater understanding of the importance of primary teeth [8].

The preference for oral hygiene maintenance methods reveals that most parents in rural areas favor the use of toothpaste and toothbrushes. A similar finding was observed by Mishra et al. [4], which is encouraging, as it aligns with modern dental recommendations. However, a significant portion still relies on traditional practices like using neem sticks, which underscores the importance of oral health education in promoting evidence-based practices [7,9]. The study identifies oral prophylaxis as the most preferred dental treatment. This preference may reflect a positive focus on preventive care. However, the limited preference for restorative treatments and stainless steel crowns indicates potential gaps in awareness of the importance of timely treatment for primary teeth. The present study correlates with the findings of Al-Batayneh et al. [10].

It has become apparent that there exists a significant lack of awareness among parents regarding the significance of primary teeth and specific dental issues. A substantial proportion of parents do not recognize the significance of primary teeth or have limited knowledge about specific dental issues. These findings underscore the urgent need for targeted educational initiatives in rural areas. Patil et al. also emphasized the importance of regular oral health awareness programs in rural populations [11].

Educational programs should be designed to bridge knowledge gaps for parents in rural areas. These programs should emphasize the significance of primary teeth, the importance of regular dental checkups, and the availability of effective dental treatments for children [12]. Community-based dental health initiatives should be strengthened to provide easy access to dental care in rural areas. These initiatives can include mobile dental clinics and awareness campaigns [13,14]. Given the prevalence of traditional practices like using neem sticks, educational efforts should be culturally sensitive. Integrating traditional practices with evidence-based oral hygiene methods can promote better oral health outcomes [14,15]. Encouraging parents to participate in their child's oral hygiene routine actively is essential. Parents who understand the importance of oral health are more likely to instill good habits in their children [16,17].

The inferential statistical analysis provides significant insights into the relationships between demographic factors and oral health behaviors among rural parents. Our findings indicate that a child's gender significantly influences oral hygiene practices, suggesting a potential bias or difference in health priorities based on gender. This underscores the need for gender-sensitive health promotion strategies that cater specifically to the nuances of each gender's needs. Additionally, the age of the mother at marriage, which was significantly associated with the choice of dental treatments, highlights how early family responsibilities might shape health decision-making processes. Moreover, the correlation between parents' education levels and their knowledge about the importance of primary teeth emphasizes the critical role of educational attainment in enhancing oral health awareness. The significant relationship between family structure and treatment preferences suggests that the type of family (nuclear vs. joint) can influence dental care decisions, pointing to the need for community-based educational programs that are adaptable to different family settings. These statistically significant associations provide a compelling argument for the design of tailored interventions that consider these demographic characteristics to improve oral health outcomes in rural areas effectively.

Study limitations include potential sampling bias (the study's sample is limited to parents seeking dental consultation for their children aged two to seven years, potentially excluding those who do not seek dental care due to various reasons such as financial constraints, lack of awareness, or cultural beliefs). This self-reporting bias could lead to a biased representation of parental preferences in rural areas. The study focuses on a specific geographical area (rural regions of Uttar Pradesh) and may not be representative of parental preferences and knowledge in other rural areas or urban settings, limiting the generalizability of the findings, potential confounding factors, the cross-sectional design's inability to establish causality, and the need for more in-depth qualitative exploration of underlying reasons or barriers influencing parental preferences and knowledge in rural areas. Addressing these limitations in future research could enhance understanding and improve oral health outcomes for children in rural populations.

## Conclusions

This research sheds light on the preferences and knowledge of parents regarding oral hygiene maintenance and dental treatment for children in rural populations. It underscores the need for comprehensive educational interventions tailored to the unique sociocultural context of rural areas. By addressing the identified knowledge gaps and promoting evidence-based oral health practices, we can contribute to improved oral health outcomes for children in rural communities, ultimately enhancing their overall well-being. Further research and collaborative efforts between healthcare providers, educators, and policymakers are essential to effecting positive change in the oral health landscape of rural populations.
